# Stanford Emerson Chaille

**Published:** 1908-07

**Authors:** Lewis S. McMurtry

**Affiliations:** Louisville, Ky. The Atherton


### Stanford Emerson Chaille.

AN ADDRESS DELIVERED ON THE OCCASION OF THE CHAILLE JUBILEE, AT NEW ORLEANS, MAY 19, 1908.

By LEWIS S. McMURTRY, A. M., M. D., Louisville, Ky.

*Ladies and Gentlemen*: To the student of Roman history the age of Augustus is both notable and distinguished. In that golden era all that was greatest in science, art and literature was apparently achieved. To the future historian of the natural and physical sciences the present age will be marked by hitherto unequaled accomplishments, and will ever remain the golden era of discovery and practical achievement in those sciences. Ours is indeed the Augustan age of science. It has chained the lightning, girdled the earth with a net-work of wires, transmits both the spoken and written word from house to house, from continent to continent, utilises the unseen currents of the atmosphere, and has made everything organic and inorganic subservient to its convenience and enjoyment. An age of such marvelous works must ever be preëminent in the history of the world's progress.

The science of medicine is closely allied to the natural and physical sciences, as is indicated by that old and familiar appellation "physician," meaning in its broadest sense a student of nature. Indeed the sciences of biology, chemistry and physics supply the very foundation of the science and art of medicine. Hence, it is only a natural sequence that an age of such wonderful achievement in these fundamental sciences should be equally prolific in the advancement of all the departments of medical science. The researches of Pasteur in biology provided the basis for the era-making discoveries of Lister, Semmelweiss and Koch; the marvelous practical application of electricity found its expression in medicine in the Röntgen ray, and the study of insect life culuminated in the conquest of the greatest plague of modern times. All these great achievements, revolutionising within a decade established teaching and accepted practice, have made modern medicine and surgery the crowning glory of pure and applied science. So brilliant and salutary are the results of modern medicine in its application to the cure and prevention of disease, that even a brief mention of them would transcend the time limit of this occasion.

To the superficial observer it would appear that our predecessors from the age of Hippocrates down to the latter half of the nineteenth century had lived in vain, and that all true science emanated from our own times. But it is not so. All knowledge is progressive and marches by slow degrees. Moreover, all knowledge is correlated, and a single observation of nature's work, apparently insignificant, may in time lead to important discoveries. The achievements of medicine in our time are marvelous indeed, but the foundations of medical science were laid deep and strong before the beginning of the nineteenth century. Bichat and Malgaigne preceded Virchow and Koch: Sydenham, Louis and Trousseau prepared the way for Von Ziemssen and Osler: Ambroise Paré, Hunter and Brodie were needed for the development of Velpeau, Paget, Syme, Gross, Billroth, Sims, Langenbeck, and Lister. These men, and many others, working through the centuries, developed and established the scientific methods of study and investigation out of which sprang the marvels of our own boasted age. The work of medical science is enormous. It is incessant. It is continuous. It consists of research, development, demonstration, experimentation, application and diffusion. It requires an army of workers, directed by many master minds. Our age is fortunate in enjoying the perfection in brilliant results by modern masters of the devoted labors of our predecessors throughout the centuries.

During this epoch-making half-century the worthy recipient of the honors of this occasion has devoted his superb talents and energies to this great profession. Stanford Emerson Chaillé was born in Mississippi; educated in Massachusetts, receiving the A. B., degree from Harvard University in 1851. Coming immediately from Harvard to this University, he received the degree of Doctor in Medicine here in 1853. In 1858 he was elected Demonstrator of Anatomy in the Medical Department of the University of Louisiana. In 1860 and 1861 he pursued post-graduate studies in Paris, devoting himself especially to physiology in the laboratory of Claude Bernard. In those days Paris was universally recognised as the medical center of the civilised world, and Bernard was the greatest physiologist of his age. At the outbreak of the Civil War, Dr. Chaillé returned to his adopted home in New Orleans, and at once entered the Southern Army, first as a private and later as Surgeon and Medical Inspector serving until the close of the war in 1865. He returned immediately to New Orleans and to the University, taking up his labors as a teacher in the Medical Department. In 1866 he returned to Paris and resumed his studies with Professor Bernard. It was here that he received his inspiration for his future brilliant career as a teacher of physiology. In 1867 he returned to New Orleans and entered upon his life work as Professor of Physiology and Pathological Anatomy, which chair he has occupied continuously until the present time.

During all these years Dr. Chaillé has been one of the foremost citizens of Louisiana. With an active mind, stored with scientific and practical knowledge; with unbounded energy; with an innate love of justice and truth; deeply imbued with civic pride and patriotism, his influence has been wielded incessantly for the upbuilding and advancement of this city and state. His fund of knowledge is not only broad, but exceptionally accurate and thorough. Both by nature and training he is possessed of the scientific spirit, which searches always for truth. An authority of national repute in state medicines and sanitary science, his services have ever been invoked and given to protect the health and lives and welfare of the people. In 1885, by appointment of the President of the United States, his expert knowledge on State Medicine was utilised in work of national scope. The clauses relating to State Medicine in the Louisiana Constitution of 1879, and the excellent laws embraced in the code of this state were formulated by him and enacted through his influence.

As I know Dr. Chaillé best as a teacher it is appropriate that I speak of him particularly as such. This is, after all, his chief life work. His preëminence as a teacher is due to several causes. His mental temperament, as well as his early training, fitted him especially for the important branch of medical science to which he has devoted his life. A pupil of Bernard, he received in early life the training so essential. He is always master of the subject under consideration. To my mind, however, his power comes from that greatest accomplishment a teacher can possess, an unusual gift,—the power to impart knowledge. His enthusiasm regarding his subject is contagious; his earnestness inspires, and his capacity to simplify complex subjects is unsurpassed. His personality is intimately associated in the minds of his pupils with his teaching. I have heard many teachers in medicine; I have visited many schools of medicine in both this country and Europe, besides having been a teacher for twenty years, and I am pleased to say that I have never known any one so *impressive*, or who imparts to the student so much knowledge as a permanent possession, as does the honored master of this occasion.

It is a great and noble work to apply at the bedside and in the operating room the life-giving resources of medicine and surgery. But how much greater to altogether prevent the existence of a mortal disease than to cure single cases of such a malady! Likewise, how transcendent is the work of annually preparing one hundred men to send forward on a life of usefulness in the cause of humanity. Such is the function of the capable and conscientious teacher of medicine.

Dr. Chaillé: In behalf of the three thousand alumni of the Medical Department of the University to which you have given so many years of your useful life, and as one of your old pupils, I bring you our congratulations and affectionate greetings. The new building of the medical department on the University Campus, the new Richardson Memorial, was appropriately dedicated by you this afternoon. Its foundation cannot be stronger than the ties of appreciation and affection which exist for you in this community, nor deeper than the respect and esteem in which you are held by the medical profession of America. The very life of the medical department is in its alumni. The world, perhaps rightly, judges by results; and the professional public demands an ever advancing standard of proficiency. The present high standing and assured progress of this department is the result of the labors of yourself and your colleagues during the years of your devoted service. A majority of your distinguished colleagues of the present faculty have been your pupils, likewise the junior instructors; and throughout the length and breadth of the land hundreds who have followed your teaching are in the honorable pursuit of their profession. The influence of such a life work is incalculable. You have indeed erected a monument more durable than brass. I congratulate you upon all that has gone before, and also upon the present. It is all beautiful. It is as it should be: "honor to whom honor is due."

THE ATHERTON.

---

### METHOD OF THE SPREAD OF YELLOW FEVER.

COL. W. C. GORGAS, Chief Sanitary Officer of the Panama Canal Commission, Ancon, Canal Zone, (*Medical Record*, June 27, 1908,) after his careful studies of the origin of yellow fever and its methods of spreading at Havana and in Panama concludes that as long as the number of stegomyia mosquitos is above a certain point the disease will spread, but when, by fumigation and isolation of the sick, it falls below that figure the disease will cease to spread and come under control. A certain number of infected mosquitos must escape fumigation but seem to do no harm. He gives arguments from his experience to support the view advanced.

